# Steel surface defect segmentation with SME-DeeplabV3+

**DOI:** 10.1371/journal.pone.0329628

**Published:** 2025-08-14

**Authors:** Haiyan Zhang, Zining Zhao, Yilin Liu, Jiange Liu, Tingmei Ma, Kexiao Wu, Zhiwen Zhuang, Jiajun Wang

**Affiliations:** 1 College of Computer and Software Engineering, Huaiyin Institute of Technology, Huaian, China; 2 Huai’an Power Supply Branch, State Grid Jiangsu Electric Power Co., Ltd., Huaian, China; Sunway University, MALAYSIA

## Abstract

Accurate segmentation of steel surface defects is crucial for ensuring steel quality. This paper presents a steel surface defect segmentation method based on SME-DeepLabV3+ to improve the accuracy and efficiency of segmentation. First, StarNet is adopted as the backbone network, whose unique star operation can achieve efficient transformation from low-dimensional space to high-dimensional features, enhancing the model’s ability to capture steel defect features and accurately distinguish between normal and defective areas. Second, the ELA module is introduced, which is based on an efficient local attention mechanism and uses different sizes of convolution kernels for multiscale analysis of feature maps. During training, it adaptively initializes the weights of convolutional layers and introduces a dynamic threshold adjustment mechanism to adjust thresholds in real time according to the defect conditions of training batches, reducing missed detections and false positives. Finally, we integrate the MSAA module from CM-UNet, whose multiscale attention mechanism can dynamically adjust attention allocation based on defect size, avoiding detection omissions or misjudgements caused by size differences. The experimental results show that the improved model performs excellently in steel surface defect segmentation tasks, significantly improving accuracy and efficiency compared with traditional methods. The mIoU, precision, and MPA evaluation metrics increased by 1.65%, 2.19%, and 0.36%, respectively, providing more effective technical support for steel quality inspection. The combination of StarNet with the MSAA and ELA modules effectively enhances the performance of semantic segmentation models in steel defect detection while reducing computational resource requirements. The code is available at https://github.com/Eric-863/SME-main/tree/main.

## 1. Introduction

Steel, as a cornerstone of modern industry, is widely and deeply applied in critical fields such as the construction, machinery manufacturing, and automotive industries. In the construction field, the surface quality of steel directly affects the structural stability and safety of buildings. High-quality steel surfaces ensure tight bonding with materials such as concrete, effectively bearing the weight of buildings and resisting natural disasters. In the machinery manufacturing industry, minor flaws on the steel surface can cause serious malfunctions during equipment operation, affecting production efficiency and increasing maintenance costs. In the automotive industry, the surface quality of steel directly impacts the aesthetic appearance, corrosion resistance, and service life of vehicles. Therefore, precise and efficient detection and segmentation of steel surface defects are crucial for ensuring smooth industrial production and improving product quality.

In steel surface defect segmentation, numerous researchers have actively explored and achieved a series of results. Sandler et al. proposed MobileNetV2 [1,2], which uses a lightweight architecture and shows certain advantages in scenarios with stringent computational resource requirements, especially in real-time detection for mobile devices. However, when applied to steel surface defect segmentation, its limited feature extraction capability makes it difficult to capture subtle defect features, affecting the accuracy of steel quality assessment. When applied to steel surface defect segmentation, the limitations of its feature extraction capability become apparent. Steel surface defects are diverse and complex, and MobileNetV2 often has difficulties capturing the subtle features of small defects, resulting in insufficient segmentation accuracy. Yu et al. [3] improved DeepLabv3+ [4] by designing a lightweight complex-valued Xception as the backbone network, reducing the number of convolution kernels in each layer of the entry flow, middle flow, and exit flow and lowering model complexity while ensuring performance; although it reduces model complexity, it has poor adaptability to special defect features when handling diverse defects, leading to incomplete segmentation. Du et al. [5] introduced D − S evidence theory and the CRF framework, further considering spatial context information, and effectively improved segmentation performance through multimodule combinations. However, the model structure is complex, is computationally intensive, has slow training and inference speeds, and cannot meet real-time detection needs in actual production. Liu et al. [6] proposed a residual spatial pyramid pooling module that captures multiscale contextual information through atrous convolutions with different dilation rates. However, the attention allocation for defects of different scales is inflexible and easily ignores small defects or loses the details of large defects when handling defects with significant size differences, which affects the integrity and accuracy of segmentation. Wang et al. [7] integrated class feature attention mechanisms into DeepLabv3 + , enabling the model to focus more precisely on different object categories when processing high-resolution images, which improves semantic segmentation accuracy. However, in steel surface defect segmentation, owing to the influence of steel material properties and blurred defect boundaries, distinguishing between defects and normal regions is not accurate enough and is easily affected by background noise. Hu [8] et al. proposed a cross-dimensional feature attention aggregation network for the recognition of clouds and snow in high-resolution satellite images. In the algorithm design, innovative structures such as a context attention aggregation module and a multi-scale strip convolution module were adopted. There are certain similarities in feature processing and model optimization ideas between this network and steel surface defect segmentation.Xiao et al. [9] proposed a weighted attention mechanism that connects with U-Net through residual connections and alleviates gradient vanishing and degradation problems, thereby improving network feature extraction capabilities. Although it alleviates gradient issues, the accuracy in segmenting small and complex defects is still unsatisfactory.

The steel surface defect segmentation method based on SME-DeepLabV3 + proposed in this paper aims to address the shortcomings of the aforementioned methods. StarNet [10] is selected as the backbone network, and its unique star operation is used to achieve efficient conversion and extraction of features from low-dimensional to high-dimensional spaces, compensating for the insufficient feature extraction capabilities of networks such as MobileNetV2 and sensitively capturing the complex features of small defects. The integration of the MSAA [11] and ELA [12] modules, which are based on multiscale attention mechanisms and efficient local attention mechanisms, respectively, enables the precise capture and analysis of defects of different sizes and enhances model adaptability through adaptive weight initialization and dynamic threshold adjustment mechanisms, solving issues related to unreasonable attention allocation and low detection sensitivity in other methods when handling defects of different scales, effectively avoiding missed detections and false positives, and comprehensively improving the segmentation accuracy and performance of the model to provide more reliable technical support for steel surface defect segmentation.

The main contributions of this paper are as follows:

(1) StarNet as the Backbone Network: DeepLabV3 + is selected as the basic architecture, and StarNet is used as the backbone network. The principle of the unique star operation in StarNet is deeply analysed, and its internal mechanism for efficiently transforming features from low-dimensional space to high-dimensional space is explored. This transformation can strengthen the model’s ability to capture steel defect features, which significantly enhances the perception accuracy of various defects and thus achieves a precise distinction between normal steel areas and defect areas and improved segmentation accuracy.
(2) MSAA Module: The multiscale attention aggregation (MSAA) module is integrated from CM-UNet into the improved model. The working mechanism of the multiscale attention mechanism in the MSAA module, which can dynamically adjust attention allocation on the basis of defect size and effectively avoids detection omissions or misjudgements caused by size differences, is explored in detail. Through this method, the detection sensitivity and segmentation accuracy of the model are significantly improved, enabling it to identify different-sized steel surface defects more comprehensively and accurately.
(3) ELA Module: The ELA module, which uses 3 × 3 and 7 × 7 convolution kernels for multiscale analysis of feature maps, is introduced based on an efficient local attention mechanism. Small convolution kernels capture the fine details of tiny defects, whereas large convolution kernels focus on the overall morphology of large defects. During training, the module adaptively initializes the weights of the convolutional layers based on dataset characteristics. Moreover, a dynamic threshold adjustment mechanism is introduced to adjust thresholds in real time according to the defect conditions of training batches, reducing the number of missed detections and false positives.

### 2. Related work

Before delving into the steel surface defect segmentation method based on SME-DeepLabV3 + , a detailed analysis of the key technologies involved is crucial; this not only helps us understand the advantages and limitations of existing technologies but also provides a solid theoretical foundation for proposing subsequent improvements. The following section elaborates on two key aspects, classic models and core network architectures, laying a solid foundation for building more efficient steel surface defect segmentation models.

### 2.1. Overview of the DeepLabV3 + Model

DeepLabV3 + is a classic model in semantic segmentation that combines dilated convolutions and encoder−decoder structures, achieving excellent results in image semantic segmentation tasks. The model expands the receptive field without increasing the computational load through dilated convolutions, thereby obtaining richer contextual information; the encoder−decoder structure effectively extracts and restores features, achieving precise classification of pixels of different categories in images. In steel surface defect segmentation, DeepLabV3 + can relatively accurately segment larger area defects, but its feature extraction capability has limitations when dealing with small defects, requiring improvement in segmentation accuracy.

### 2.2. Encoder−Decoder Network

**Fig 1:**
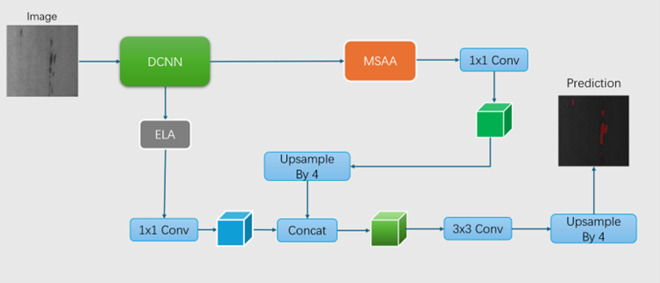
Schematic of the improved DeepLabv3 + model. The ASPP module is replaced with a multiscale attention aggregation (MSAA) mechanism, and the ELA module is added to the shallow feature extraction part to enhance feature extraction.

Owing to its outstanding performance and wide applicability, the encoder−decoder network occupies a key position in computer vision and has been deeply integrated into many critical tasks, such as human pose estimation, object detection, and semantic segmentation. This network architecture consists of two core parts: the encoder module and the decoder module. Among them, the encoder module effectively captures higher-level semantic information of images while gradually reducing feature maps, providing key semantic support for subsequent processing; the decoder module is responsible for restoring abstract semantic information into specific image segmentation results through step-by-step recovery of spatial information, realizing the conversion from semantic understanding to specific region division of images. The research model innovatively designs based on the classic encoder−decoder architecture. The specific process is as follows: the input steel surface image first passes through the DCNN module to extract basic features. In one branch, the feature map processed by the MSAA undergoes channel adjustment and feature fusion through a 1 × 1 convolution. In another branch, the feature map output by the DCNN [13] is first processed via the ELA module, then undergoes a 1 × 1 convolution and is concatenated with the upsampled feature map to fuse hierarchical features, thereby improving the accuracy of segmenting subtle defects. The concatenated feature map undergoes further feature extraction through a 3 × 3 convolution, followed by upsampling to achieve feature map fusion. In this process, the MSAA module dynamically allocates attention based on defect size, reducing the number of missed detections and false positives; the ELA module plays a role in shallow feature extraction, enhancing feature extraction capabilities. The fused feature map is upsampled to restore dimensions and outputs prediction results. The improved model architecture is shown in Fig 1.

## 3. Methodology

To address the precision and efficiency bottlenecks faced in steel surface defect segmentation tasks, this study innovatively proposes a systematic improvement strategy. Starting from optimizing the core architecture of the model, a new StarNet network is selected to replace the traditional backbone network, leveraging its unique star operation to achieve efficient feature conversion and extraction. Simultaneously, two key modules, MSAA and ELA, are integrated based on multiscale attention mechanisms and efficient local attention mechanisms, respectively, to capture and analyse defects of different sizes precisely. Adaptive weight initialization and dynamic threshold adjustment mechanisms are employed to enhance model adaptability. These improvements work synergistically to comprehensively enhance model performance, providing robust technical support for accurate and efficient steel surface defect segmentation. The following sections delve into the specific details and implementation principles of each improvement method.

### 3.1. StarNet as a backbone network

In terms of model architecture innovation, this study selects StarNet as the backbone network, replacing traditional network architectures to fundamentally enhance the model’s ability to extract features of steel surface defects. As a novel type of neural network architecture, the core advantage of StarNet lies in its unique star operation. The star operation, through specific convolution operations and feature fusion methods, can achieve the efficient transformation of features from low-dimensional space to high-dimensional space. Specifically, in a single layer of a neural network, the star operation is typically expressed as ${(W}_{1}^{T}X+B_{1})*{(W}_{2}^{T}X+B_{2})$. For ease of analysis, the weight matrix and bias can be combined and simplified to ${(W}_{1}^{T}X)*{(W}_{2}^{T}X)$. Taking the single-output-channel transformation and single-element input scenario as an example, $w_{1}$, $w_{2}$, and $x\in {\mathbb{R}}^{(d+1)\times 1}$ (d is the number of input channels) are defined after a series of derivations as follows:


$$w_{1}^{T}x*w_{2}^{T}x$$ (1)

$$\begin{array}{c} =\left(\sum {\mathrm{\backslash nolimits}}_{i=1}^{d+1}w_{1}^{i}x^{i}\right)*\left(\sum {\mathrm{\backslash nolimits}}_{j=1}^{d+1}w_{2}^{j}x^{j}\right) \end{array}$$ (2)

$$\begin{array}{c} =\sum {\mathrm{\backslash nolimits}}_{i=1}^{d+1}\sum {\mathrm{\backslash nolimits}}_{j=1}^{d+1}w_{1}^{i}w_{2}^{j}x^{i}x^{j} \end{array}$$ (3)

$$=\alpha_{\left(1,1\right)}x^{1}x^{1}+\ldots +\alpha_{\left(4,5\right)}x^{4}x^{5}+\ldots +\alpha_{\left(d+1,d+1\right)}x^{d+1}x^{d+1}$$ (4)

The above Formula (4) contains a total of $\frac{(d+2)(d+1)}{2}$ terms, among which . The star operation can be extended to $\frac{(d+2)(d+1)}{2}\approx {\left(\frac{d}{\sqrt{2}}\right)}^{2}$ (when d≫2), the combinations of different terms are nonlinearly related to x, meaning that they represent independent implicit dimensions. Therefore, the star operation can efficiently compute in d-dimensional space while achieving feature representation in an approximate implicit-dimensional feature space of ${\left(\frac{d}{\sqrt{2}}\right)}^{2}$, significantly expanding the feature dimensions without additional computational overhead.

**Fig 2:**
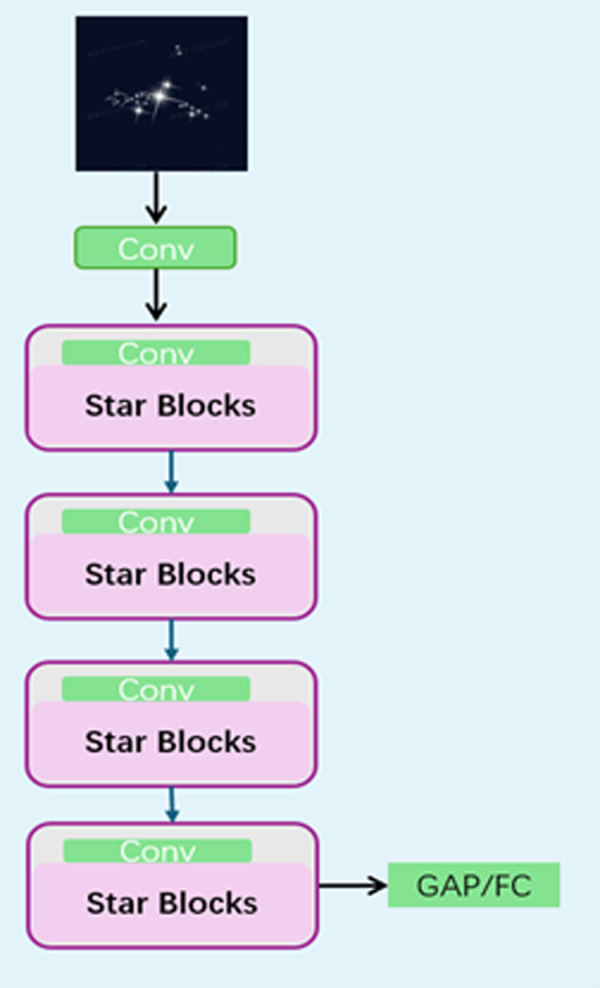
StarNet structure diagram. StarNet follows the traditional hierarchical network, directly using convolutional layers to downsample the resolution, doubling the number of channels at each stage, and repeating multiple star blocks to extract features.

The StarNet architecture consists of multiple sequentially arranged star blocks. Within each star block, meticulously designed convolution operations deeply mine potential relationships in different dimensions of the input feature map and extract and reorganize features precisely. This unique design makes StarNet highly sensitive to weak, complex features in minute steel surface defects, converting these easily overlooked features into highly representative high-dimensional features. The StarNet structure diagram is shown in Fig 2.

**Fig 3:**
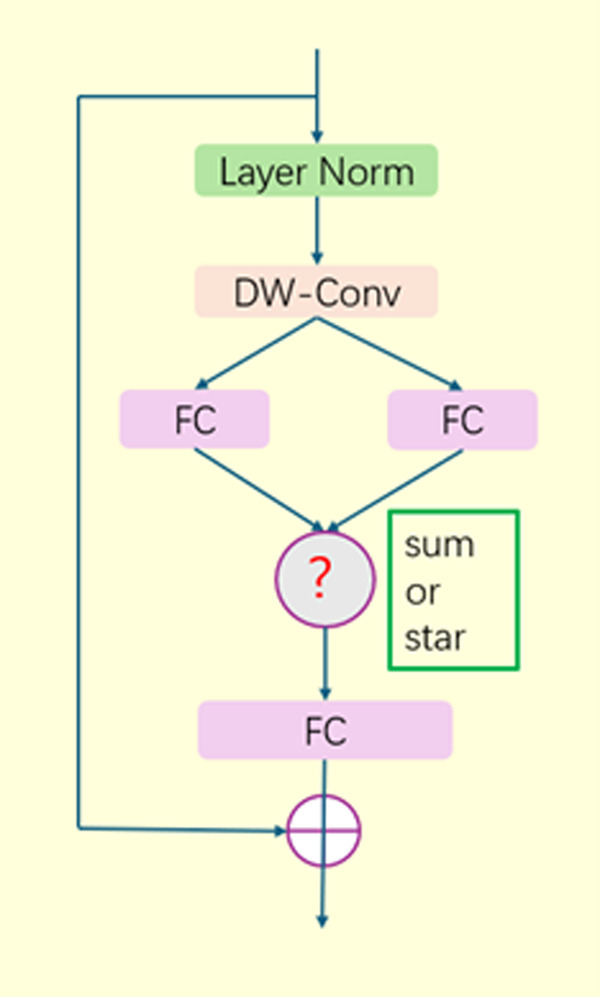
Structure diagram of the star blocks. A comparison between the star operation and the summation operation shows that the star operation consistently outperforms the summation operation, especially in narrower networks; this is attributed to its ability to map inputs to high-dimensional space without expanding the network width.

The star operation in star blocks consistently outperforms summation, especially in narrower width networks; this is attributed to its ability to map inputs to high-dimensional space without expanding the network width. The transformation mechanism of the star operation enhances StarNet’s feature extraction capability, making it particularly suitable for capturing subtle features in complex data. The structure of the star blocks is shown in Fig 3.

### 3.2. MSAA module

This study incorporates the MSAA module from CM-UNet into the model, providing a new perspective and significantly improving steel surface defect segmentation. The core innovation of the MSAA module lies in its advanced multiscale attention mechanism, which is specifically designed to address the complex characteristics of steel surface defects of varying sizes.

The module uses different sizes of convolution kernels, such as small 3 × 3 convolution kernels, which focus on the fine details of small defects and do not miss any subtle feature changes, and large 7 × 7 convolution kernels, which focus on the overall shape of large defects and grasp the macrofeatures of defects. Through this multiscale collaborative analysis, the MSAA module can comprehensively and meticulously capture the key features of defects of different sizes; it intelligently initializes the weights of internal convolutional layers based on the distribution characteristics of defect types and sizes in the steel surface defect dataset. This innovation allows the MSAA module to adapt quickly to the feature patterns of defects of different scales in the early stages of training, paying more targeted attention to various defects and effectively improving training efficiency and effectiveness.

**Fig 4:**
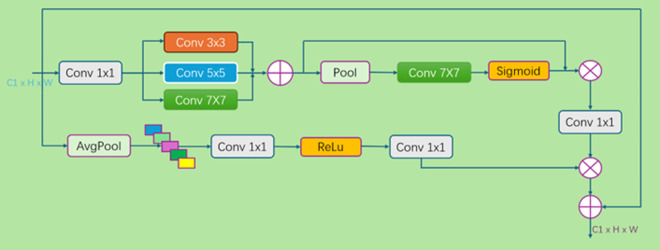
Structural diagram of the multiscale attention aggregation (MSAA) module. This module uses different sizes of convolution kernels, such as 3 × 3, 5 × 5, and 7 × 7, to perform multiscale analysis on feature maps, with small convolution kernels capturing fine details of small defects and large convolution kernels focusing on the overall shape of large defects.

The dynamic threshold adjustment mechanism of the MSAA module can monitor the average size and number of defects in the current training batch in real time, precisely adjusting the threshold based on these dynamically changing parameters. When batches with more small defects are encountered, the threshold is automatically lowered to increase the model’s sensitivity to small defects and ensure that no minor flaw is missed; when large defects dominate, the threshold is increased in a timely manner to ensure the integrity of segmentation for large defects and avoid overlooking the overall structure due to excessive focus on details. Through this dynamic and intelligent threshold adjustment strategy, the MSAA module effectively avoids detection omissions and misjudgements, greatly improving the model’s detection sensitivity to small defects and the segmentation integrity for large defects, significantly enhancing the model’s performance in steel surface defect segmentation tasks. The MSAA structure diagram is shown in Fig 4.

### 3.3. ELA module

**Fig 5:**
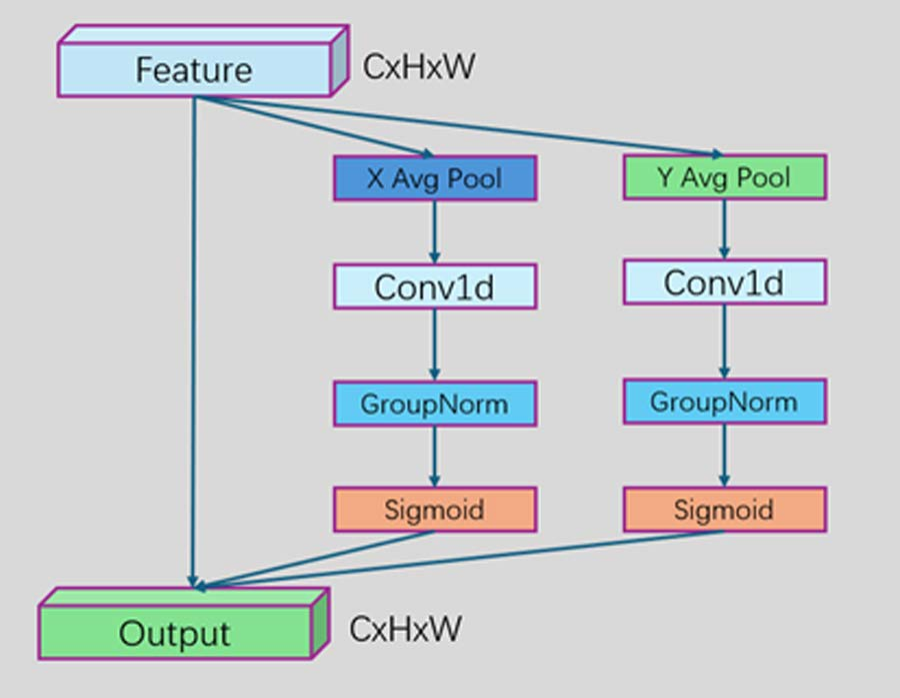
ELA module structure diagram. The structure of the ELA module and the process of performing multiscale analysis on feature maps via convolution kernels and related operations is shown.

The ELA module introduced in this study represents an breakthrough in enhancing the performance of the steel surface defect segmentation model. The ELA module is based on an efficient local attention mechanism, using 3 × 3 and 7 × 7 convolution kernels to perform deep multiscale analysis on feature maps. Small convolution kernels focus on capturing the fine details of small defects, presenting clear features such as subtle edges and unique textures of small defects like a magnifying glass; large convolution kernels focus on the overall shape of large defects, grasping key information such as shape and range from a macroperspective. Through the collaboration of small and large convolution kernels, the ELA module can comprehensively and meticulously analyse defect features of different scales, providing rich feature information for accurate segmentation. The ELA module also has a dynamic threshold adjustment mechanism, significantly enhancing the detection sensitivity and segmentation accuracy for defects of different sizes. The ELA structure diagram is shown in Fig 5.

## 4. Experimental evaluation

To comprehensively and objectively verify the effectiveness of the improved DeepLabv3 + -based steel surface defect segmentation method, a series of experiments was conducted, the improved model was evaluated from multiple dimensions, and it was compared with other relevant models.

### 4.1. Dataset

**Fig 6:**
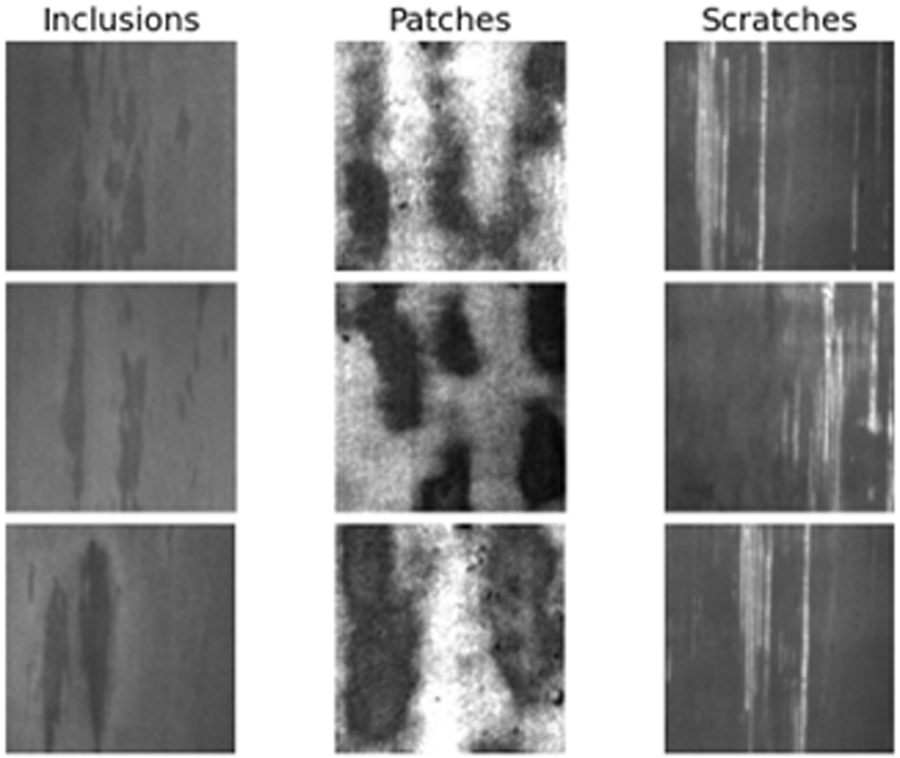
Sample images of steel defects. From left to right are sample images of inclusions, patches, and scratches.

The NEU_Seg steel defect detection dataset was selected. This dataset covers various types of steel surface defects and contains abundant defect samples and corresponding annotation information, which can be used to test the model’s ability to segment different defects in complex steel surface backgrounds. This dataset was compiled and developed by institutions such as Vicomtech, aiming to provide a high-quality dataset for steel surface defect detection. In steel production, surface defect detection is crucial for ensuring product quality. The NEU_Seg dataset contains many steel surface images that cover various common defect types such as patches, inclusions, and scratches, as well as normal steel surface areas as background. The dataset includes steel surface images under different scene and lighting conditions, as well as samples of various defect types and severity levels, to fully test the generalization ability and robustness of the model, allowing the trained model to adapt to various complex situations in practical applications. Fig 6 shows sample images of three typical steel defects.

This article uses the VOC format for training. Before training, the image files are placed in the JPEGImages folder under the VOC2007 folder in the VOCdevkit folder. The label files are placed in the SegmentationClass folder under the VOC2007 folder in the VOCdevkit folder. Specifically, there are three types of steel defect categories, totalling 3,630 steel defect images, which are divided into training and validation sets at a ratio of 9:1. Additionally, 840 images do not participate in training and are used as the testset.

### 4.2. Evaluation metrics

Mean Intersection over Union (mIoU): This metric is used to comprehensively measure the accuracy of the model in segmenting different categories. During calculation, first, the ratio of the intersection to the union between the predicted segmentation result and the ground truth annotation for each category (i.e., IoU) is determined, and the values are subsequently averaged across all categories. The closer the mIoU value is to 1, the higher the model’s segmentation accuracy. The calculation formula is as follows:


$$\begin{array}{c} mIoU=\frac{1}{N}\sum {\mathrm{\backslash nolimits}}_{i=1}^{N}IoU_{i} \end{array}$$ (5)

where N represents the total number of categories and $IoU_{i}$ is the intersection over union (IoU) for the i-th category.

Precision: This metric reflects the accuracy of the model in identifying defect regions, i.e., the proportion of truly positive samples (defect regions) among those predicted as positive by the model. Higher precision indicates fewer false positives. The calculation formula is as follows:


$$Precision=\frac{TP}{TP+FP}$$ (6)

where TP represents true positives, which is the number of defect areas correctly identified by the model, and FP represents false positives, which refers to the number of normal areas incorrectly classified as defect areas by the model.

Recall: This reflects the model’s ability to detect actual defect areas. The higher the recall is, the more actual defects the model detects and the fewer defects that are missed. The calculation formula is as follows:


$$Recall=\frac{TP}{TP+FN}$$ (7)

In the formula, FN represents false-negatives, which are the areas where defects actually exist but are not detected by the model.

The MPA, or mean pixel accuracy, is a key metric for evaluating model performance. It assesses the model’s prediction accuracy at the pixel level, which is calculated as the mean of the correct prediction ratios for each category. The calculation formula is as follows:


$$\begin{array}{c} MPA=\frac{1}{N}\sum {\mathrm{\backslash nolimits}}_{I=1}^{N}\frac{TP_{i}}{TP_{i}+FN_{i}} \end{array}$$ (8)

where N represents the total number of categories, $TP_{i}$ is the number of pixels correctly predicted for the i-th category, and $FN_{i}$ is the number of pixels in the i-th category that were misclassified into other categories. The closer the MPA value is to 1, the higher the accuracy of the model in classifying the pixels of different steel surface defects and normal regions.

### 4.3. Experimental environment

This experiment was set up on the Alibaba Cloud server platform, with Python 3.8, CUDA 11.8, an NVIDIA A10 GPU, and 30 GB of memory. The SGD optimizer was selected for the experiment, with 300 training epochs. During the experiment, the input image size was uniformly adjusted to 512 × 512. Moreover, data augmentation methods such as random flipping and rotation were used to expand the data, thereby enhancing the model’s generalizability.

### 4.4 Comparative experiment

The improved model (ours) was compared with classic semantic segmentation models such as Res-UNet,DeepLabV3 + , PSPNet, and HRNet. The performance of each model was tested on the NEU_Seg dataset, and the results are shown in Table 1.

**Table 1:** Performance comparison of the model with other semantic segmentation models.

Method	mIoU (%)	Precision (%)	MPA (%)	Recall (%)
Res-UNet [9]	81.68	89.48	89.55	89.55
PSPNet(MobileNetV2) [14,15]	81.64	90.22	88.74	88.74
PSPNet(ResNet50) [16]	82.25	90.59	89.18	89.18
HRNet(hrnetv2_w18) [17,18]	83.04	90.45	90.26	90.26
HRNet(hrnetv2_w32) [19]	83.48	90.67	90.63	90.63
HRNet(hrnetv2_w48) [20]	83.33	91.67	90.43	90.43
DeepLabV3+(MobileNetV2) [21]	87.22	90.53	95.76	95.76
DeepLabV3+(Starnet+ASPP)	88.77	91.99	96.05	96.05
DeepLabV3+(Starnet+M)	88.52	91.83	95.92	95.92
DeepLabV3+(Starnet+ASPP+E)	88.57	91.72	95.97	95.97
DeepLabV3+(Starnet+ASPP+M)	88.39	91.96	95.62	95.62
DeepLabV3+(Xeception)	88.09	91.51	95.74	95.74
**DeepLabV3+(Starnet+M + E)**	**88.87**	**92.72**	**96.10**	**96.10**

**Fig 7:**
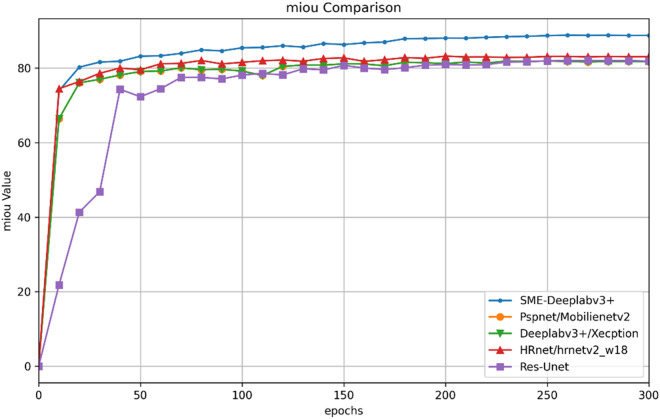
Charts comparing the mIoU values.

The comparison results show that the improved model outperforms other comparative models in terms of the mIoU, precision, MPA, and recall metrics; this demonstrates that the DeepLabv3 + model improved with the StarNet, MSAA(M), and ELA(E) modules performs better in steel surface defect segmentation tasks and can identify and segment defect areas more accurately and quickly, effectively detecting more actual defects. The mIoU comparison for the above models is shown in Fig 7.

The figure shows that the improved model outperforms the other comparative models in terms of the mIoU metric. This finding indicates that the improved model has higher segmentation accuracy for different defects and normal areas on steel surfaces, can more accurately identify and segment defect areas, and performs better in steel surface defect segmentation tasks.

### 4.5. Ablation experiment

To explore the roles of various components in the improved model, ablation experiments were designed. Using StarNet as the backbone network, the computational complexity and model size of the model with the original ASPP module and the integrated MSAA and ELA modules were compared, as shown in Table 2.

**Table 2:** Computational Complexity and Model Size of StarNet with Modules.

Backbone	ASPP	MSAA	ELA	GFLOPS	Params
StarNet	**√**	**√**	**×**	74.604 G	11.161M
	**×**	**√**	**×**	56.845 G	8.928M
	**√**	**×**	**×**	71.280 G	10.753M
	**√**	**×**	**√**	71.281 G	10.753M
	**×**	**√**	**√**	**56.845 G**	**8.928M**

Table 2 demonstrates the performance of the model that uses StarNet as the backbone network and that uses the original ASPP module and integrates the MSAA and ELA modules in terms of the GFLOPS (computational load) and Params (parameter count) metrics. Integrating the MSAA and ELA modules significantly impacts the model’s computational load and parameter count. Removing the ASPP module and integrating MSAA (i.e., “StarNet, **×**, **√**, and **×**”) reduces the GFLOPS from 74.604 G to 56.845 G and the Params from 11.161M to 8.928M, indicating that the MSAA module can effectively reduce the model’s computational complexity and scale. Adding the ELA module alone (“StarNet, **√**, **×**, and **√**”) results in relatively smaller changes in computational load and parameter count, with GFLOPS and Params values of 71.281 G and 10.753M, respectively, suggesting that the ELA module maintains model scale and computational load stability. Integrating both the MSAA and ELA modules (“StarNet, **×**, **√**, and **√**”) results in a GFLOPS of 56.845 G and Params of 8.928M, facilitating the construction of a lightweight model that balances performance and efficiency, providing better options for practical applications.

When StarNet is used as the backbone network, the experimental results show that integrating the MSAA and ELA modules improves the model’s mIoU and precision metrics. This finding indicates that the multiscale attention mechanism of the MSAA module can effectively enhance the model’s detection capability for defects of different sizes, improving segmentation accuracy. When StarNet is used as the backbone network, the overall performance of the model is better than that of MobileNetV2 and Xception, further verifying the advantage of StarNet in capturing steel defect features. The experimental results are shown in Table 3:

**Table 3:** Comparison of StarNet as the backbone network combined with modules.

Backbone	ASPP	MSAA	ELA	mIoU	Precision	MPA
StarNet	**√**	**√**	**×**	88.39%	91.96%	95.62%
	**×**	**√**	**×**	88.52%	91.83%	95.92%
	**√**	**×**	**×**	88.77%	91.99%	96.05%
	**√**	**×**	**√**	88.57%	91.72%	95.97%
	**×**	**√**	**√**	**88.87%**	**92.72%**	**96.10%**
Xecption	**×**	**√**	**√**	84.79	84.79	91.96
	**√**	**×**	**×**	88.09	91.51	95.74
MobileNetV2	**√**	**×**	**×**	87.22	90.53	95.76
	**×**	**√**	**√**	84.67	92.43	90.44

By observing the results of the ablation experiment, the model is analysed from two aspects: (1) performance indicators and (2) model parameter count and computational load:

(1) From the perspective of model performance indicators, when StarNet is used as the backbone network, the performance of the model varies with different module combinations. When the MSAA module is integrated, the mIoU and precision metrics of the model improve, indicating that its multiscale attention mechanism can effectively enhance the model’s detection capability for defects of different sizes, improving segmentation accuracy. Using the ELA module alone can also optimize the model performance to a certain extent. When both the MSAA and ELA modules are used together, the mIoU reaches 88.87%, the precision reaches 92.17%, and the MPA reaches 96.10%. Compared with using them separately, the performance improvement is more obvious, demonstrating the synergistic effect between the two modules.
(2) Comparing the model parameter count and computational load (GFLOPS) with different module combinations, the addition of the ASPP module significantly increases the model’s parameter count and computational load. For example, when StarNet is paired with the ASPP module, the parameter count and GFLOPS are significantly higher than when only the MSAA or ELA module is used. This indicates that while high model performance is pursued, computational resource limitations need to be considered comprehensively. The MSAA and ELA modules help construct lightweight and efficient steel surface defect segmentation models while ensuring a certain level of performance improvement.
(3) Table 3 shows StarNet’s distinct advantages as the sole backbone. Its star operation enables efficient low-to-high-dimensional feature transformation (Formulas 1–4), enhancing sensitivity to subtle defects. For example, StarNet+ASPP achieves 88.77% mIoU and 91.99% precision, outperforming MobileNetV2 and Xception.With MSAA and ELA, StarNet+MSAA+ELA achieves the highest metrics (88.87% mIoU, 92.72% precision). MSAA’s multiscale attention adapts to defect sizes, while ELA’s convolutions enhance local features, reducing small-defect misses and improving large-defect segmentation.Replacing ASPP with MSAA cuts GFLOPS from 74.604G to 56.845G and parameters from 11.161M to 8.928M (Table 2), balancing performance and efficiency for industrial use.

### 4.6. Experimental summary and analysis

In this experiment, a comprehensive evaluation of the improved steel surface defect segmentation model was conducted, and the results revealed that the model achieved significant performance improvements. This achievement is closely related to the three key innovations adopted by the model. The following section presents an in-depth analysis of the reasons for the improvement in the experimental results based on these innovations.

1. StarNet as the Backbone Network: The experimental results show that when StarNet is used as the backbone network, the overall performance of the model is better than that of MobileNetV2 and Xception; this is due to the unique star operation of StarNet, which can achieve the efficient transformation of features from low-dimensional space to high-dimensional space. In steel defect detection, this transformation significantly enhances the model’s ability to capture complex features of small defects. From the ablation experiment data, even without integrating the MSAA and ELA modules, the model can maintain a relatively high segmentation accuracy to some extent when StarNet is used as the backbone network, with the mIoU reaching 88.77% and the precision reaching 91.99%. This finding indicates that the powerful feature extraction capability of StarNet lays a solid foundation for subsequent precise segmentation. Its star operation expands feature dimensions without adding much computational overhead through specific convolution operations and feature fusion, enabling the model to convert subtle features of the steel surface into more representative high-dimensional features, thereby accurately distinguishing between normal and defect areas, effectively improving segmentation accuracy, and performing well in various evaluation metrics.
2. Integration of the MSAA Module: The MSAA module, which is based on a multiscale attention mechanism, plays a key role in improving model performance in the experiment. The ablation experiment reveals that after the MSAA module is integrated, the mIoU and precision metrics of the model significantly improve; this is because the module cleverly uses different sizes of convolution kernels for multiscale analysis, targeting the characteristics of large size differences in steel surface defects. The small 3 × 3 convolution kernel focuses on the fine details of small defects and does not miss any subtle feature changes; the large 7 × 7 convolution kernel focuses on the overall shape of large defects. Moreover, the MSAA module adaptively initializes weights based on the characteristics of the defect dataset, quickly adapts to the feature patterns of defects of different scale in the early stages of training, pays more targeted attention to various defects, and effectively improves training efficiency and effectiveness. Its dynamic threshold adjustment mechanism also plays a significant role. When batches with more small defects are encountered, the threshold to increase sensitivity to small defects is automatically lowered; when large defects dominate, the threshold to ensure the integrity of segmentation for large defects is increased in a timely manner. This dynamic and intelligent adjustment strategy effectively avoids detection omissions and misjudgements, greatly improves the model’s detection capability for defects of different sizes and significantly enhances the model’s performance in steel surface defect segmentation tasks, thereby improving the values of the mIoU, precision, and other evaluation metrics.
3. Introduction of the ELA Module: The ELA module, which is based on an efficient local attention mechanism, also significantly enhances the model’s performance in detecting and segmenting steel surface defects. The experimental results show that after incorporating the ELA module, the model’s detection sensitivity and segmentation accuracy are notably improved. The ELA module uses 3 × 3 and 7 × 7 convolution kernels to perform deep multiscale analysis on feature maps. Small convolution kernels capture fine details of small defects, presenting clear features such as subtle edges and unique textures of these defects; large convolution kernels focus on the overall shape of large defects and grasp key information such as shape and range from a macroperspective. Through this collaborative work of small and large convolution kernels, the ELA module can comprehensively and meticulously analyse defect features of different scales, providing rich feature information for accurate segmentation. Additionally, the ELA module has a dynamic threshold adjustment mechanism, which significantly enhances the detection sensitivity and segmentation accuracy for defects of different sizes.
4. Synergistic Effect Analysis: These three innovations do not function in isolation but rather collaborate synergistically to collectively enhance model performance. The powerful feature extraction capability of StarNet provides high-quality feature maps for the MSAA and ELA modules, enabling them to more effectively analyze and process defects of different sizes. Notably, the MSAA and ELA modules exhibit distinct yet complementary roles:
  (1) The MSAA module dynamically allocates attention across scales using its multiscale attention mechanism, prioritizing defect features based on their size (e.g., emphasizing small defects with 3 × 3 kernels and large defects with 7 × 7 kernels).
  (2) The ELA module enhances the representativeness of multiscale features at the shallow stage through 3 × 3 and 7 × 7 convolutions, capturing fine-grained details (e.g., micro-textures of scratches) and coarse-grained structures (e.g., macro-shapes of patches).

This division of labor creates a “feature enhancement-attention refinement” pipeline: ELA provides rich multiscale features as the foundation, while MSAA optimizes their utilization by suppressing background noise and focusing on scale-relevant defects. Their synergy is particularly evident in mixed defect scenarios, where ELA’s detailed feature maps allow MSAA to accurately distinguish small inclusions from large patches.

Experimental results validate this synergy: when all three innovations are integrated, the model achieves optimal performance with an mIoU of 88.87%, precision of 92.72%, and MPA of 96.10%. Compared to models using only StarNet+MSAA (mIoU: 88.52%) or StarNet + ELA (mIoU: 88.57%), the joint use of MSAA and ELA yields a 0.35%–0.30% improvement in mIoU, demonstrating that their combined effect is far greater than the sum of individual contributions. This highlights the necessity of their co-design to address the complex, multi-scale nature of steel surface defects.

**Fig 8:**
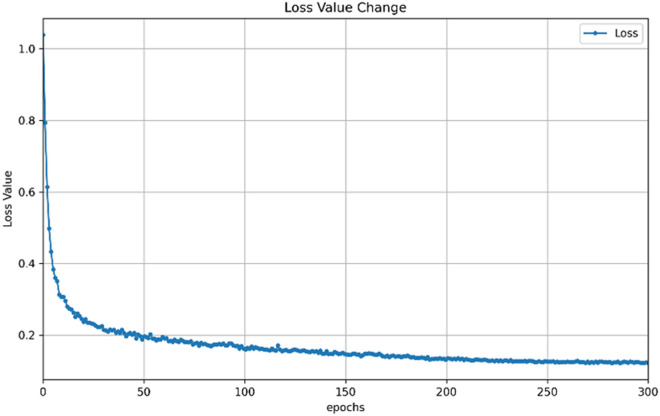
Curve of the change in model loss function.

**Fig 9:**
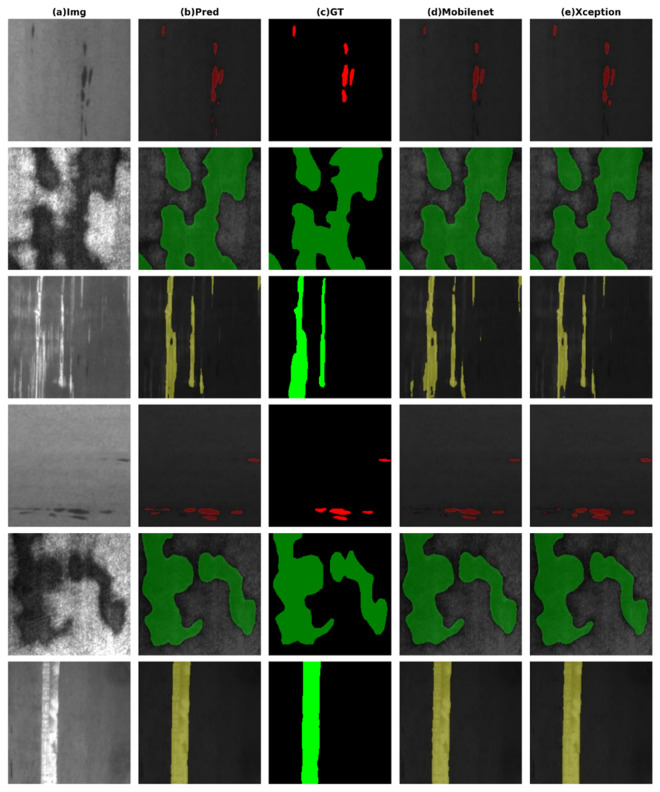
Comparative visualization of defect segmentation results (from left to right: original image, predictions of the improved model, ground truth, MobileNetV2 baseline, Xception baseline).

5. Loss Function Variation: During the training of deep learning models, the loss function is a key indicator measuring the difference between the model’s predicted results and true labels. In this study, cross – entropy loss, focal loss, and Dice loss were employed to train the model. The combination of these loss functions can effectively guide the model to optimize parameters and improve the performance of steel surface defect segmentation. The model’s loss function variation curve (Fig 8) shows a downwards trend as the number of training epochs increases. In the early stages of training, the model still explores the characteristics of steel surface defect data, and the parameters are not fully optimized, resulting in higher loss values. As training progresses, the model continuously adjusts its parameters, such as the weights involved in the star operation of StarNet and the convolution kernel weights of the MSAA and ELA modules, to better fit the data. During this process, the model’s ability to capture defect features continues to strengthen, and the difference between the prediction results and true labels gradually decreases, leading to a decline in the loss function value. The star operation of StarNet enables efficient conversion from low-dimensional to high-dimensional features, allowing the model to quickly learn key features of steel surface defects during training and providing strong support for reducing the loss value. The MSAA module dynamically adjusts attention allocation based on defect size, and the ELA module performs multiscale analysis on feature maps via different-sized convolution kernels. These two modules adaptively initialize weights and dynamically adjust thresholds during training, helping the model identify defects more accurately and further promoting a decline in loss values. When training reaches a certain number of epochs, the decline in the loss function slows and stabilizes; this indicates that the model learns most of the effective features in the current dataset, and the effect of parameter adjustments on optimizing the loss value gradually weakens. At this point, the model reaches a relatively stable state and can accurately segment steel surface defects. However, owing to the complex and diverse nature of steel surface defects, there may still be some difficult-to-learn features, preventing the loss value from decreasing further.

6. Defect Segmentation Results: As shown in Fig 9, the segmentation performance of the improved model for typical steel surface defects (inclusions, patches, and scratches) is displayed from left to right. Column (a) presents the original images, clearly showing the morphological characteristics of the defects. Column (b) demonstrates the model’s prediction results, accurately identifying the boundaries of various defects. Column (c) provides ground truth annotations as a reference, while columns (d) and (e) showcase the performance of MobileNetV2 and Xception as baseline models, respectively.

For inclusion defects (e.g., the first row of examples), the model precisely outlines the contours of inclusions, achieving high consistency with the ground truth without significant mis-segmentation or background misclassification. This is attributed to the enhanced local texture feature extraction capability of the improved model. In the case of patch-type defects (e.g., the middle row of examples), the segmentation results fully cover the defect regions with smooth and intact boundaries, indicating the model’s superior global perception capability for large-scale irregular defects. Regarding scratch detection (e.g., the bottom row of examples), the model successfully identifies fine linear scratches, even capturing subtle defects that are easily overlooked by traditional methods. This is achieved through the synergistic effect of the improved multi-scale feature fusion mechanism and small convolutional kernels.

The experiments demonstrate that the improved model outperforms the baseline models across all defect segmentation tasks. However, its performance can still be affected by complex backgrounds, necessitating further optimization of feature decoupling capabilities to enhance adaptability in industrial scenarios.

## 5. Conclusion

This work focuses on steel surface defect segmentation and innovatively proposes the SME-DeepLabV3 + segmentation method, which aims to overcome the challenges of existing technologies in terms of accuracy and efficiency, providing strong technical support for steel quality inspection. From the perspective of innovative methods, this study selects StarNet as the backbone network, whose unique star operation breaks tradition by achieving efficient conversion from low-dimensional to high-dimensional features. This characteristic greatly enhances the model’s ability to capture steel defect features, enabling it to accurately identify and distinguish between normal steel areas and defect areas and laying a solid foundation for subsequent precise segmentation. Moreover, the MSAA and ELA modules are innovatively integrated. The MSAA module, which is based on advanced multiscale attention mechanisms, can intelligently and dynamically allocate attention resources according to differences in defect sizes, effectively avoiding missed detections and misjudgements caused by different defect sizes. The ELA module uses different-sized convolution kernels to perform multiscale analysis on feature maps, adaptively initializing convolutional layer weights based on dataset characteristics and introducing a dynamic threshold adjustment mechanism. This design can adjust thresholds in real time according to the defect conditions in training batches, further reducing the probability of missed detections and false positives and significantly improving the model’s detection performance.

In summary, the improved steel surface defect segmentation method achieves a good balance between segmentation accuracy and computational resource consumption and provides an efficient and reliable technical solution for steel quality inspection with extremely high practical application value.
